# Robust order representation is required for backward recall in the Corsi blocks task

**DOI:** 10.3389/fpsyg.2014.01285

**Published:** 2014-11-10

**Authors:** Katsuki Higo, Takehiro Minamoto, Takashi Ikeda, Mariko Osaka

**Affiliations:** ^1^Graduate School of Human Sciences, Osaka UniversitySuita, Japan; ^2^Department of Psychology, Graduate School of Letters, Kyoto UniversityKyoto, Japan; ^3^Department of Adaptive Machine Systems, Graduate School of Engineering, Osaka UniversitySuita, Japan

**Keywords:** working memory, spatial, Corsi blocks task, serial order memory, recall order

## Abstract

The storage and processing of spatial information is done by spatial working memory. To measure spatial working memory, the Corsi blocks task, which separates the memory into two types, forward and backward, is often used. Although it had been thought that backward recall requires more of the executive function than forward recall, some studies have shown otherwise. Here, we focused on the spatial and sequential aspects of the Corsi blocks task to investigate cognitive processes by dissociating forward and backward recall. We used a dual task method (serial articulatory suppression or spatial tapping as the secondary task) and analyzed two kinds of errors (position error and order error) to investigate cognitive performance during the forward and backward recall. We ran two experiments: in experiment 1, we employed the standard Corsi blocks task, and in experiment 2, we employed the modified Corsi blocks task in order to prevent verbal strategies. We found that spatial tapping affected both forward and backward recall, while serial articulatory suppression increased the number of order errors in the backward condition. These results indicate that stronger order representation is required for backward recall in the Corsi blocks task.

## INTRODUCTION

### SPATIAL WORKING MEMORY

Working memory is a limited capacity form of the human memory system that involves the temporary storage and manipulation of information. To explain how working memory functions, Baddeley and Hitch (1974) proposed the working memory model, which consists of three components: the central executive, the visuospatial sketch pad and the phonological loop. The central executive and visuospatial sketch pad are the major constituents of spatial working memory, as they act as the control system of working memory and the storage of visual and spatial information, respectively.

### CORSI BLOCKS TASK

**FIGURE 1:**
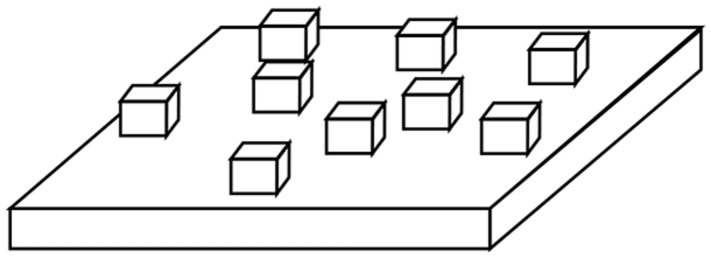
**Apparatus of the Corsi blocks task.** The experimenter taps blocks sequentially and the participant is required to recall the block sequence.

Spatial working memory is frequently assessed by the Corsi blocks task (Milner, 1971). The task requires participants to store and reproduce a sequence of block locations (**Figure 1**) and consists of two phases: encoding and recall. During the encoding phase, the experimenter taps the blocks to present a sequence of target blocks to the participant. In the recall phase, participants are required to reproduce the sequence in the same order as presented. The task has two conditions, forward and backward, which have different demands on memory. In the forward condition, participants are required to reproduce the sequence in the same order as presented, whereas in the backward condition they must reproduce it in the reverse order. A similar task is included in the Wechsler memory scale-III (Wechsler, 1997), which is widely used to measure individual intelligence. Despite these experimental systems, the cognitive processes underlying working memory are not well understood, especially with regards to differences in the two conditions.

It has been proposed that the forward condition measures the visuospatial short-term memory span, whereas the backward condition measures that and the executive function (Carlesimo et al., 1994; Cherry et al., 1996; Hester et al., 2004). Similarly, in the digit span test, the forward condition is thought to measure passive verbal short-term memory, while the backward condition measures short-term memory and the executive function. Indeed, the backward digit span is shorter than the forward digit span (Wilde and Strauss, 2002; Wilde et al., 2004; Kessels et al., 2008). Additionally, the dorsolateral prefrontal cortex was observed to be more active during the backward digit span than the forward digit span, which suggested the former depends more on executive function (Gerton et al., 2004). However, numerous studies have found no differences in behavioral performance between the two conditions when using the Corsi blocks task (Berch et al., 1998; Wilde and Strauss, 2002; Kessels et al., 2008). Thus, it is unclear whether there are cognitive differences between the demands of the forward and backward recall in the Corsi blocks task.

### SPATIAL PROCESSING AND SEQUENTIAL PROCESSING IN CORSI BLOCKS TASK

Mammarella and Cornoldi (2005) suggested that executive function is not a critical factor for dissociating backward and forward recall. Instead, they argued that differences could be attributed to different degrees of dependency on spatial processing, having found that children who had disabled visuospatial learning had difficulty in performing backward recall in the Corsi blocks task but showed normal backward recall in the digit span task. Although the children did not have general difficulties in executive function, they had difficulty in performing the backward Corsi blocks task. However, Vandierendonck et al. (2004) showed that performance in the forward and backward conditions did not differ when spatial processing was disrupted, indicating that both recalls may require similar spatial processing.

In addition to spatial processing, sequential processing is another important factor for the Corsi blocks task, because serial recall is mandatory. Given that forward and backward recall may depend on the sequential processing in different manners (e.g., backward recall might recruit more processing than forward recall), we investigated cognitive processes upon dissociating forward and backward recall by disrupting spatial processing function or sequential processing function in the Corsi blocks task.

To study working memory, a dual task method was used, where participants were required to perform a primary and secondary task simultaneously. The purpose of this system was to evaluate performance of the primary task when disrupted by the secondary task. This method has shown that a verbal secondary task disrupts a verbal primary task but not visuospatial primary task, while a visuospatial secondary task disrupts a visuospatial primary task but not verbal primary task (Baddeley, 2007), which suggests that the verbal memory and visuospatial memory systems are dissociated in working memory. In a dual task method, articulatory suppression is often used as the verbal secondary task (Murray, 1968), and spatial tapping is often used as the spatial secondary task (Farmer et al., 1986). Articulatory suppression requires that participants continuously utter a word such as “the,” while spatial tapping requires that participants tap some points sequentially with their fingers.

Although verbal secondary tasks are considered not to interfere with visuospatial task, Jones et al. (1995) and Depoorter and Vandierendonck (2009) reported that serial articulatory suppression interferes with serial spatial memory. In the former work, a dot memory task was employed. This task required participants to remember the order of dots that appeared on a display sequentially and differs from the Corsi blocks task in that there are no placeholders, as only one dot was presented at a time. In the recognition phase, all dots in the stimulus sequence were re-presented simultaneously at their original positions. Participants were required to point to each dot in the order in which they were originally presented. At the same time, participants were required to perform two kinds of articulatory suppression, steady-state and changing-state, during the encoding and retention phases. In the steady-state articulatory suppression condition, participants were required to utter the syllable “bee” repeatedly, and in the changing-state articulatory suppression condition, they were required to utter the alphabetic sequence “a” to “g.” More errors were found in the changing-state articulatory suppression condition than the steady-state articulatory suppression and control conditions. The authors explained that the changing-state articulatory suppression disrupted spatial order memory, because both the spatial memory task and the changing-state articulatory suppression contained cues about the serial order, while the steady-state articulatory suppression was not serial and therefore did not have this effect.

Depoorter and Vandierendonck (2009) also investigated the effects of serial and non-serial verbal secondary tasks on serial visuospatial primary tasks. They employed spatial tasks as primary tasks, in which participants were required to remember the order of the spatial position sequence of five squares presented sequentially. The task demanded serial order memory. For the secondary task, a letter order memory task, in which participants were required to remember the order of a five-letter sequence, and a letter item memory task, in which participants were required to remember five-letters presented simultaneously, were used. In the spatial primary and letter order memory tasks, participants needed to remember the order only (they needed not remember the spatial position or letters themselves). In this experiment, a trial started with the stimuli presentation for the primary spatial order memory task followed by the presentation of the stimuli for the secondary verbal task (letter order memory task or letter items memory task). The letter stimuli presentation was followed by the recognition phase of the task. Finally, the recognition phase of the primary spatial task was administered. The letter order memory task disrupted the spatial order memory task, while the letter items memory task did not disrupt the spatial order memory task. The authors interpreted these results as possible evidence for a modality-independent order coding system such that secondary tasks that demand order processing disrupt spatial order memory.

Thus, because a secondary task that demands order processing, even if it is verbal modality, disrupts remembering the order information of the spatial task, we employed serial articulatory suppression (changing-state articulatory suppression) as a secondary task to disrupt serial processing in the Corsi blocks task. Furthermore, because the Corsi blocks task demands both position memory and order memory, we also employed spatial tapping as a secondary task to spatial memory.

### OBJECTIVE OF THE PRESENT STUDY AND EXPERIMENTAL PROCEDURE

The present study aimed to specify which cognitive processes dissociate forward and backward recall in the Corsi blocks task. Specifically, we focused on spatial processing and sequential processing.

In previous studies, the accuracy of the performance in the Corsi blocks task was mainly analyzed and other behavioral indices were less attended. However, those indices may provide a clue on which cognitive processes differentially recruited the forward and backward recall. There are two errors in the Corsi blocks task: position error and order error. Position error, which appears when spatial processing is disturbed, occurs when participants select a block that was not highlighted during the encoding phase. On the other hand, order error, which is observed when sequential processing is impaired, occurs when participants select a block that was presented but at an incorrect serial position. Paying more attention to those behavioral indices, we investigated the effects of secondary tasks on the Corsi blocks task.

The present study consisted of two experiments. In experiment 1, a standard computer-based Corsi blocks task was administered, and participants performed forward and backward recall under three different conditions: spatial tapping, serial articulatory suppression and control (no secondary task). The set-size of the number of items to be remembered was also manipulated (3, 5, and 7 blocks). In experiment 2, a layout of blocks was varied across trials in order to prevent verbal coding of the block locations. Because an effect of the secondary task was observed only in the seven-items condition in experiment 1, we fixed the set-size to seven items in experiment 2.

### EXPERIMENTAL HYPOTHESIS

Using the above experimental procedure, we tested the following hypothesis. If spatial processing critically differs between forward and backward recall in the Corsi blocks task, then spatial tapping will increase the number of position errors in the backward condition but have no effect on the forward condition. On the other hand, if sequential processing plays a critical role in dissociating forward recall from backward recall, then serial articulatory suppression will increase the number of order errors in the backward condition but have no effect on the forward condition.

## EXPERIMENT 1

### METHOD

#### Participants

Twenty-four right-handed volunteers (university students and staff; 11 male, 13 female) were recruited. Their age varied between 19 and 33, with a mean age of 22.7. The experiment was approved by the ethics committee at the Graduate School of Human Sciences, Osaka University. Our institutional review board did not demand written consent when the experiment was carried out. However, we explained our experiments to the participants such that they understood the conditions and were welcome to quit whenever they wanted. All participants consented via e-mail as an agreement of participation.

#### Apparatus

The stimulus control was regulated by Tobii Studio 2.2.8 software (Tobii Technology AB, Danderyd, Sweden). The participants were seated with their heads in a chin rest, which kept the visual distance at about 59 cm.

#### Design

The experiment had three repeated measure factors: recall order (two levels: forward and backward), secondary tasks (three levels: control, serial articulatory suppression, and spatial tapping), and sequence length of items (three levels: 3, 5, and 7). A within-participants design was used. In total, 18 conditions were prepared. There were 144 trials performed (eight for each condition). The recall order and secondary tasks were counterbalanced across participants, and the sequence length was always increased from three to seven.

#### Materials and procedures

**FIGURE 2:**
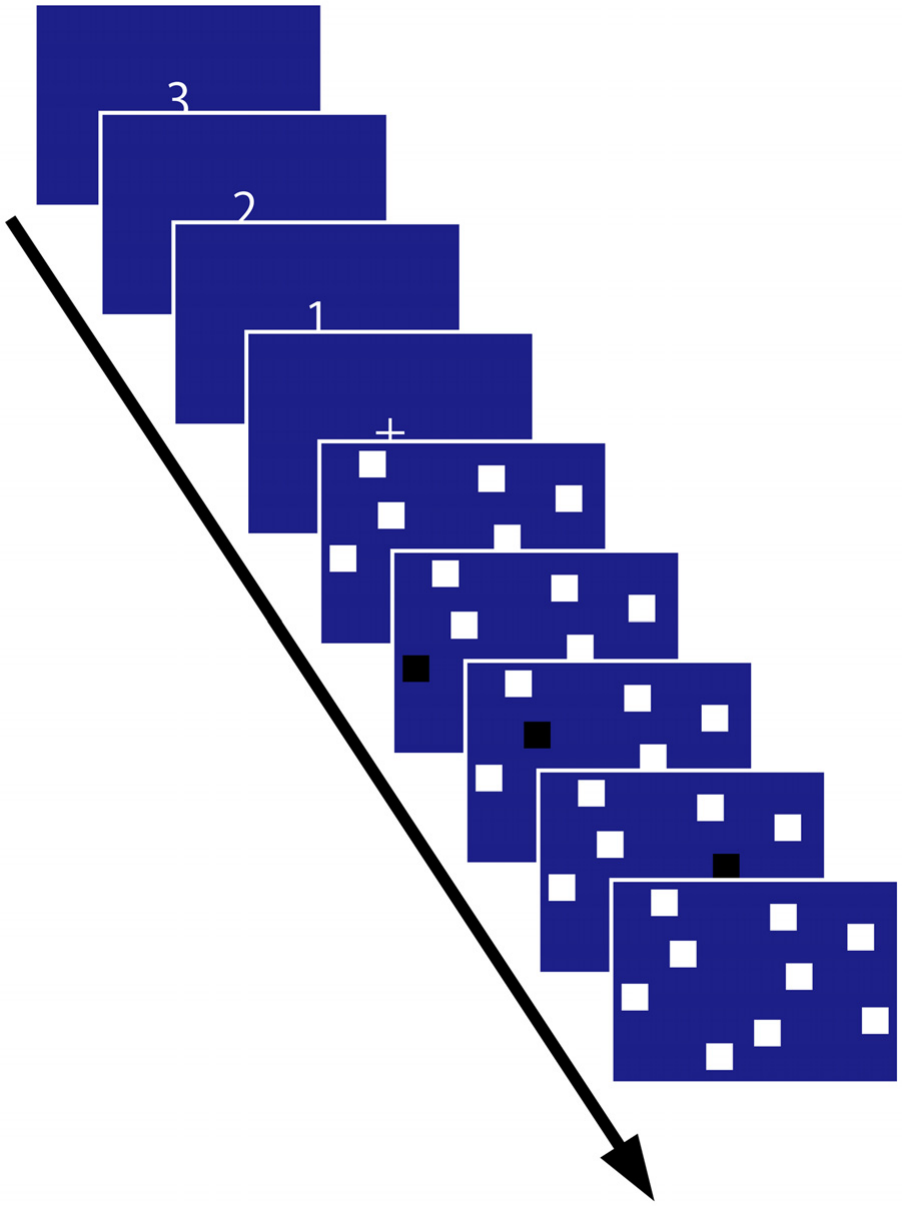
**Experimental time course.** The block sequence was presented in the encoding phase, and the participant recalled the sequence immediately afterward. This figure shows the sequence length 3 condition.

The Corsi blocks task was adapted for computerized presentation on a 17-inch TFT monitor. White squares 30 mm × 30 mm large were used as the nine placeholders and placed irregularly on a blue background (**Figure 2**). The block sequence was projected by changing the color of squares to black sequentially for 1 s in random order, and there was no inter-block time.

A 3 sec countdown commenced each trial. Next, a white fixation cross was presented for 2 sec followed by the appearance of the nine white blocks on the screen. The target stimulus sequence was presented sequentially 2 sec after presentation of the nine white blocks. Immediately after presenting the target items, the participant was required to repeat the sequence by clicking the squares using a mouse.

The secondary task was given while participants encoded the spatial items. For serial articulatory suppression, participants were required to repeat a four-sound sequence (“i,” “ro,” “ha,” “ni”) continuously at a rate of about two per second. For spatial tapping, participants were instructed to tap the four corners of a rectangle board (11 cm × 14 cm) placed on the table in front of them with their index finger of the left hand in a clockwise direction at a pace of two corners per second. In the dual task condition, the secondary task began after the 3 sec countdown and was continuous during the presentation of the blocks sequence. No secondary task was performed in the recall phase or during any of the control condition.

The experiment started with an explanation of the Corsi blocks task. Each of the conditions started with three practice trials (the sequence length of each practice trial was three).

### DATA ANALYSIS

All statistical analyses were done with R version 2.15.1 (R Development Core Team, Statistics Department of the University of Auckland). Greenhouse-Geisser correction was applied when appropriate.

## RESULTS

### CORRECT TRIALS

**FIGURE 3:**
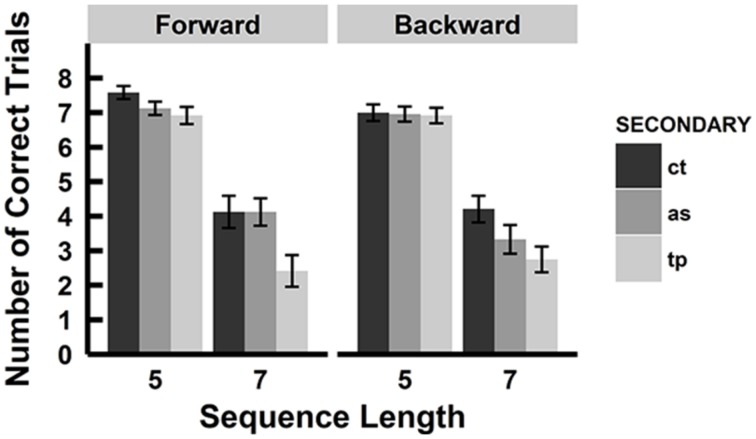
**Number of correct trials.** Number of correct trials in each condition. The interaction between the secondary tasks and the sequence length was significant. There were significant differences between the control and spatial tapping condition and between the serial articulatory suppression condition and the spatial tapping condition at sequence length 7 across the forward and backward conditions. A simple-main effect of the secondary tasks was not significant at sequence length 5. There was no effect of the serial articulatory suppression. ct, control condition; as, serial articulatory suppression condition; tp, spatial tapping condition. Error bars represent SE.

We defined correct trials as those when participants recalled all blocks in the correct order. We removed data for a sequence length of 3 from the analysis, because all participants performed the task perfectly in the forward spatial tapping condition and the backward serial articulatory suppression, resulting in a SD of zero. **Figure 3** shows the number of correct trials in each condition. We compared the average number of correct trials of all participants between each condition. A 2 (recall order: forward vs. backward) × 3 (secondary tasks: control vs. serial articulatory suppression vs. spatial tapping) × 2 (sequence length: 5 vs. 7) repeated ANOVA on the scores revealed a significant main effect of the secondary tasks [*F*(1.72, 39.52) = 10.37, *p*< 0.001, $\eta_{p}^{2}$ = 0.31] and sequence length [*F*(1, 23) = 232.87, *p* < 0.001, $\eta_{p}^{2}$ = 0.91]. A significant interaction was found between the secondary tasks and sequence length [*F*(1.45, 33.42) = 6.38, *p* < 0.01, $\eta_{p}^{2}$ = 0.22], and no significant interactions was found between the recall order and the secondary tasks [*F*(1.86, 42.69) = 1.99, *p* = 0.15, $\eta_{p}^{2}$ = 0.08] nor between the recall order and the sequence length [*F*(1, 23) = 0.14, *p* = 0.70, $\eta_{p}^{2}$ = 0.01], and no significant three-way interaction was found [*F*(1.71, 39.24) = 2.54, *p* = 0.10, $\eta_{p}^{2}$ = 0.10]. There was no significant main effect of recall order [*F*(1, 23) = 1.05, *p* = 0.32, $\eta_{p}^{2}$ = 0.04]. Further analysis for the main effect of the secondary tasks by Shaffer’s Bonferroni test showed significant differences between the control and spatial tapping conditions (*p* < 0.001, *r* = 0.77) and between the serial articulatory suppression and spatial tapping conditions (*p* < 0.05, *r* = 0.50), and no significant difference between the control and serial articulatory suppression conditions (*p* = 0.18, *r* = 0.28). Subsidiary analyses for the interaction between the secondary tasks and sequence length showed that a significant simple-main effect of secondary tasks was observed at sequence length 7 [*F*(1.36, 31.31) = 10.29, *p* < 0.01, $\eta_{p}^{2}$ = 0.31], while not at sequence length 5 [*F*(1.82, 41.93) = 2.31, *p* = 0.12, $\eta_{p}^{2}$ = 0.09]. Further analyses for the simple main effect of the secondary tasks at sequence length 7 showed that there were significant differences between the control and spatial tapping conditions (*p* < 0.001, *r* = 0.84) and between the serial articulatory suppression and the spatial tapping conditions (*p* < 0.01, *r* = 0.52), but no significant difference between the control and serial articulatory suppression conditions (*p* = 0.33, *r* = 0.20) across the forward and backward conditions. There were significant differences between the sequence lengths 5 and 7 in each of the secondary tasks conditions [control, *F*(1, 23) = 119.46, *p* < 0.001, $\eta_{p}^{2}$ = 0.84; serial articulatory suppression, *F*(1, 23) = 89.20, *p* < 0.001, $\eta_{p}^{2}$ = 0.80; spatial tapping, *F*(1, 23) = 119.97, *p* < 0.001, $\eta_{p}^{2}$ = 0.90].

### POSITION ERRORS

Position error was defined as the selection of a block that was not highlighted during the encoding phase. We compared the average number of blocks recalled in the wrong position in each condition, focusing on sequence length 7, because only this length showed a main effect of secondary tasks when comparing the number of correct trials. There were eight trials in each condition. First, we calculated the average number of order errors by each participant in each trial. For example, in eight trials, if a participants made four order errors, then his/her average number of order errors in this condition was 0.5 (=4/8). In this way, we calculated the average number of order errors in each trial for all participants. Then, we calculated the average of the average number of order errors for all participants in each condition and compared this number between conditions. The average number of position errors for all participants was compared using a 2 (recall order) × 3 (secondary tasks) ANOVA.

A significant main effect of the secondary tasks was found [*F*(1.82, 41.77) = 5.45, *p* < 0.01, $\eta_{p}^{2}$ = 0.19]. Further analyses for the main effect of the secondary tasks by Shaffer’s Bonferroni test showed significant differences between the control and spatial tapping conditions (*p* < 0.01, *r* = 0.58), and between the serial articulatory suppression and spatial tapping conditions (*p* < 0.05, *r* = 0.46). No significant difference was observed between the control and serial articulatory suppression conditions (*p* = 0.94, *r* = 0.02). There was no significant main effect of recall order [*F*(1, 23) = 0.62, *p* = 0.44, $\eta_{p}^{2}$ = 0.03] and no significant interaction between the recall order and the secondary tasks [*F*(1.38, 31.77) = 2.28, *p* = 0.13, $\eta_{p}^{2}$ = 0.09].

### ORDER ERRORS

**FIGURE 4:**
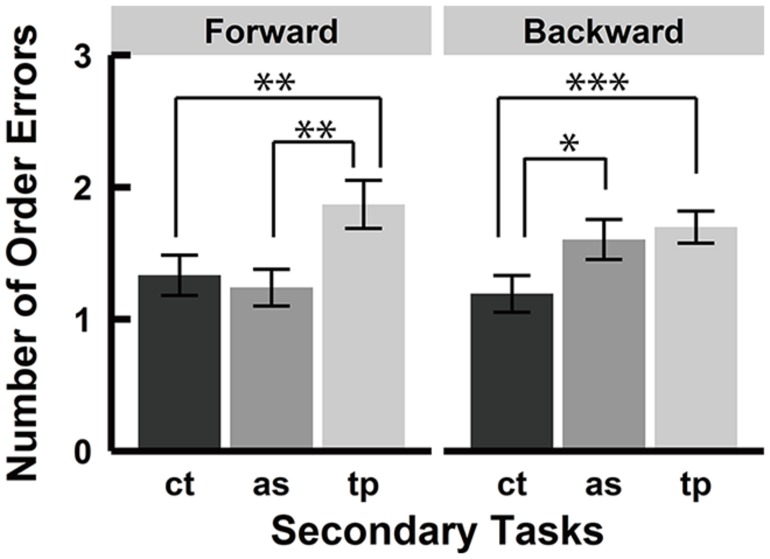
**Number of order errors.** Average number of order errors in each secondary task condition. There was a main effect of the secondary task and a significant interaction between the recall order and the secondary task. Further analyses of the main effect showed significant differences between the control and spatial tapping conditions and between the serial articulatory suppression and spatial tapping conditions. Multiple comparisons for the interaction showed significant differences between the serial articulatory suppression and spatial tapping conditions and between the control and spatial tapping conditions in the forward condition, and significant differences between the control and spatial tapping conditions and between the control and serial articulatory suppression conditions in the backward condition. (*p < 0.05, **p < 0.01, ***p < 0.001). ct, control condition; as, serial articulatory suppression condition; tp, spatial tapping condition.

Order error was defined as the errors in which participants selected a block that was presented in the encoding phase but at the wrong sequence position. **Figure 4** shows the average number of order errors by all participants in each condition. Analysis of the order error at sequence length 7 revealed a significant main effect of the secondary task [*F*(1.66, 38.22) = 9.18, *p* < 0.01, $\eta_{p}^{2}$ = 0.29] and a significant interaction between recall order and secondary tasks [*F*(1.99, 45.69) = 4.77, *p* < 0.05, $\eta_{p}^{2}$ = 0.17]. There was no significant main effect of the recall order [*F*(1, 23) = 0.03, *p* = 0.87, $\eta_{p}^{2}$ = 0.00] Further analysis for the main effect of the secondary tasks by Shaffer’s Bonferroni test showed significant differences between the control and spatial tapping conditions (*p* < 0.001, *r* = 0.75) and between the serial articulatory suppression and spatial tapping conditions (*p* < 0.05, *r* = 0.46), but no significant difference between the control and serial articulatory suppression conditions (*p* = 0.23, *r* = 0.25). Further analysis of the interaction showed that there were simple main effects of the secondary tasks in both forward and backward conditions [forward, *F*(1.93, 44.5) = 8.71, *p* < 0.001, $\eta_{p}^{2}$ = 0.27; backward, *F*(1.68, 38.63) = 6.14, *p* < 0.01, $\eta_{p}^{2}$ = 0.21]. There were significant differences in the forward condition between the control and spatial tapping conditions (*p* < 0.01, *r* = 0.58) and between the serial articulatory suppression and spatial tapping conditions (*p* < 0.01, *r* = 0.60), and in the backward condition between the control and spatial tapping conditions (*p* < 0.001, *r* = 0.67) and between the control and serial articulatory suppression conditions (*p* < 0.05, *r* = 0.46). There were no significant differences between the control and serial articulatory suppression conditions in the forward condition (*p* = 0.55, *r* = 0.13) nor between the serial articulatory suppression and spatial tapping conditions in the backward condition (*p* = 0.59, *r* = 0.11).

## DISCUSSION

The present research aimed to explore the cognitive processes underlying the Corsi blocks task. We focused on cognitive differences between the forward and backward condition in terms of spatial processing and sequential processing. The results showed that while spatial tapping increased both position and order errors in the forward and backward conditions, serial articulatory suppression selectively increased order error in the backward condition. Given that the serial articulatory suppression affects the memory representation of serial order, the present results indicate that the backward recall of the Corsi blocks task requires stronger order representation, whereas spatial representation appears equivalent across the forward and backward conditions.

### CORRECT TRIALS

Number of correct trials was inversely related to sequence length (number of items), with no difference between the forward and backward conditions, which is consistent with previous studies (Wilde and Strauss, 2002; Vandierendonck et al., 2004; Kessels et al., 2008). Concurrent spatial tapping disrupted the forward and backward recall performance, while serial articulatory suppression did not. There was no interaction between the recall order and secondary tasks. These results are consistent with one experiment (Experiment 3) in Vandierendonck et al. (2004), in which spatial tapping impaired both forward and backward recall while articulatory suppression did not.

### POSITION ERROR AND ORDER ERROR

Position error increased under spatial tapping but not under serial articulatory suppression in both the forward and backward conditions. The results indicate that forward and backward recall in the Corsi blocks task depended on common visuospatial storage at a similar magnitude. On the other hand, order error increased only in the backward condition under the serial articulatory suppression. Given that the serial articulatory suppression interfered with sequential representation, the selective impairment in the backward recall condition may indicate that backward recall requires stronger sequential representation in comparison to forward recall. A concurrent spatial tapping increased the number of order errors in both forward and backward condition. This result may be because of the spatial tapping’s interference on the integration of the position and the order information.

In experiment 1, order error was selectively increased in the backward condition when a concurrent serial articulatory suppression was given. We interpreted this result as evidence of a stronger order representation being required to perform backward recall in the Corsi blocks task. However, the selective effect of the serial articulatory suppression might be due to a verbal strategy employed by the participants. Specifically, participants might verbally code block positions (“top right,” “bottom left,” and so on), and the serial articulatory suppression might prevent participants from using the strategy especially in the backward condition. To remove this possibility, we varied the block positions across trials in experiment 2 and tested how the concurrent serial articulatory suppression affected forward and backward recall. Additionally, we analyzed the performance of secondary tasks to examine the trade-off between the primary and secondary tasks in the forward and backward conditions. The analysis was performed to exclude the possibility that poor performance of the serial articulatory suppression in the forward recall condition might contribute to fewer order errors.

## EXPERIMENT 2

The block positions were varied in each trial to prevent a verbal strategy, such as a numbering of the block positions.

### METHOD

The stimuli and procedures were mostly the same as those used in experiment 1 except that the positions of the blocks were varied in each trial. The positions of the placeholders in each trial were set as follows. The 1280 × 1024 pixel display was partitioned into invisible 3 × 3 cells of 400 × 400 pixels (the invisible cells overlapped with each other). A placeholder was presented in each cell, which was set at a random location in each cell. The positions of the placeholders were adjusted so as not to overlap each other.

#### Participants

Twenty-one right-handed volunteers (university students; 12 male, 9 female) were recruited. Their age ranged from 20 to 27, with a mean age of 22.9. One participant mentioned that she could use both hands equally. She usually writes with the left hand and manipulates a computer mouse with the right hand. In this experiment, she was asked to manipulate a mouse with the right hand and perform spatial tapping with the left hand like the other participants. The experiment was approved by the ethics committee of the Graduate School of Human Sciences, Osaka University. All participants gave their informed consent in accordance with the Department of Human Sciences, Osaka University.

#### Apparatus

Stimulus control and response collection were regulated by Presentation 16.3 software (Neurobehavioral systems, San Francisco, CA, USA).

#### Design

The experiment had two repeated measure factors: the recall order (forward and backward) and the secondary tasks (control, serial articulatory suppression, and spatial tapping). We removed the sequence lengths 3 and 5 from experiment 2, because secondary task effects were observed only at sequence length 7 in experiment 1. There were 48 trials performed (eight for each condition). The recall order and secondary tasks were counterbalanced across participants.

#### Material and procedures

Materials and procedures were the same as those used in experiment 1 except that the positions of the blocks were varied in each trial. Secondary tasks were given in the same manner as experiment 1. We used an USB ten keypad to record spatial tapping performance. Participants were instructed to hit the four keys of the keypad (“0,” “7,” “9,” “.”) in a clockwise direction. The distance between “0” key and “7” key is about 5.8 cm and between “7” key and “9” key is about 3.8 cm. Each of the conditions started with three practice trials (the sequence length of each practice trial was five).

### DATA ANALYSIS

All statistical analyses were done with R version 2.15.1 (R Development Core Team, Statistics Department of the University of Auckland). Greenhouse-Geisser correction was applied when appropriate.

## RESULTS

We excluded two participants’ data from analyses because one participant showed an extremely low number of correct trials in the control of the backward condition (2 SDs away from the mean). The other participant showed an extremely large number of order errors in the control of the backward recall condition (2 SDs away from the mean). We used a 2 (order recall: forward vs. backward) × 3 (secondary tasks: control vs. serial articulatory suppression vs. spatial tapping) repeated ANOVA and Shaffer’s Bonferroni test for all multiple comparisons.

### SECONDARY TASKS’ PERFORMANCE

We compared the performance of serial articulatory suppression and spatial tapping between the forward and backward conditions. We counted the errors of the secondary tasks. In the serial articulatory suppression, we defined “skip,” “unclear pronunciation” and “halting speech” as errors. In the spatial tapping, we defined “skip,” “reverse” and “double (participant hit same key twice)” as errors. We compared the average of number of errors in eight trials between the forward and backward conditions.

For the serial articulatory suppression errors, a paired *t*-test revealed no significant difference between the forward and backward conditions [*t*(36) = 1.24, *p* = 0.22, *r* = 0.20; **Table 1**].

**Table 1:** Number of serial articulatory suppression’s errors.

	Mean	SD
Forward	0.79	1.32
Backward	0.37	0.68

Regarding the spatial tapping errors, a paired *t*-test revealed no significant difference between the forward and backward conditions [*t*(36) = 1.05, *p* = 0.30, *r* = 0.17; **Table 2**].

**Table 2:** Number of spatial tapping’s errors.

	Mean	SD
Forward	0.63	1.26
Backward	1	0.88

### CORRECT TRIALS

**FIGURE 5:**
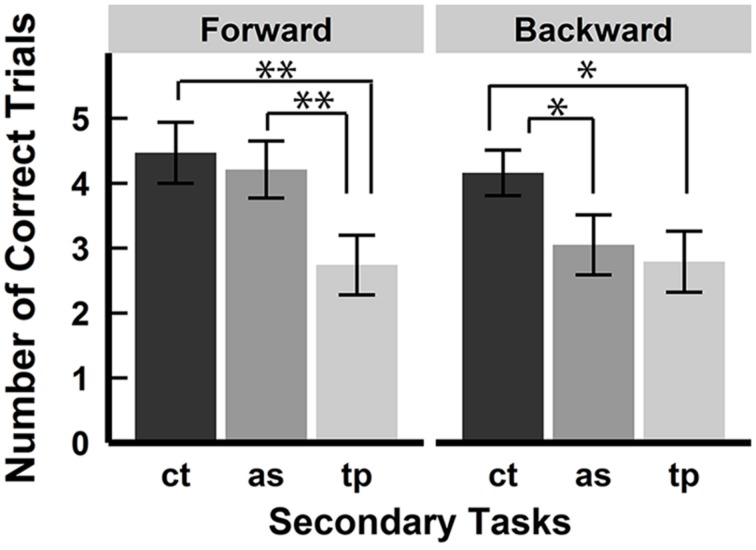
**Number of correct trials.** Number of correct trials in each secondary task condition. A significant main effect of the secondary task was observed and there were significant differences between each secondary task. Although there was no significant interaction between the recall order and the secondary tasks, multiple comparisons revealed that significant differences between the control and spatial tapping condition were observed in both forward and backward conditions, while there was a significant difference between the control and serial articulatory suppression conditions in only the backward condition. (*p < 0.05, **p < 0.01). ct, control condition; as, serial articulatory suppression condition; tp, spatial tapping condition.

**Figure 5** shows the mean and SE of the number of correct trials in each condition. A 2 × 3 repeated ANOVA on the scores revealed a significant main effect of the secondary tasks [*F*(1.96, 35.25) = 16.80, *p* < 0.001, $\eta_{p}^{2}$ = 0.48]. There was no significant main effect of the recall order [*F*(1, 18) = 1.76, *p* = 0.20, $\eta_{p}^{2}$ = 0.09], and no interaction was found between the factors of the recall order and the secondary task [*F*(1.9, 34.27) = 1.51, *p* = 0.24, $\eta_{p}^{2}$ = 0.08]. Further analysis for the main effect of the secondary task showed significant differences between the control and spatial tapping conditions (*p* < 0.001, *r* = 0.80), the serial articulatory suppression and spatial tapping conditions (*p* < 0.01, *r* = 0.59), and the control and serial articulatory suppression conditions (*p* < 0.05, *r* = 0.54). Although the interaction was not significant, we performed a simple main effect test and multiple comparisons to examine whether the same effect of the secondary tasks in experiment 1 was obtained. A simple main effect test revealed that there was a significant main effect of the secondary task in the forward [*F*(1.84, 33.09) = 8.64, *p* < 0.01, $\eta_{p}^{2}$ = 0.32] and backward conditions [*F*(1.98, 35.62) = 5.36, *p* < 0.01, $\eta_{p}^{2}$ = 0.23]. Multiple comparisons showed that there were significant differences between the control and spatial tapping conditions (*p* < 0.01, *r* = 0.68) and between the serial articulatory suppression and the spatial tapping conditions (*p* < 0.01, *r* = 0.56), but no significant difference between the control and serial articulatory suppression conditions (*p* = 0.52, *r* = 0.15) in the forward condition. There were significant differences between the control and spatial tapping conditions (*p* < 0.05, *r* = 0.61) and between the control and serial articulatory suppression conditions (*p* < 0.05, *r* = 0.50), but no significant difference between the serial articulatory suppression and spatial tapping conditions (*p* = 0.57, *r* = 0.13) in the backward condition.

### POSITION ERRORS

The average number of position errors was compared using a 2 × 3 ANOVA. This analysis revealed no significant main effects [recall order, *F*(1, 18) = 0.37, *p* = 0.55, $\eta_{p}^{2}$ = 0.02; secondary tasks, *F*(1.74, 31.33) = 1.05, *p* = 0.35, $\eta_{p}^{2}$ = 0.06] or interaction [*F*(1.93, 34.7) = 1.76, *p* = 0.19, $\eta_{p}^{2}$ = 0.09].

### ORDER ERRORS

**FIGURE 6:**
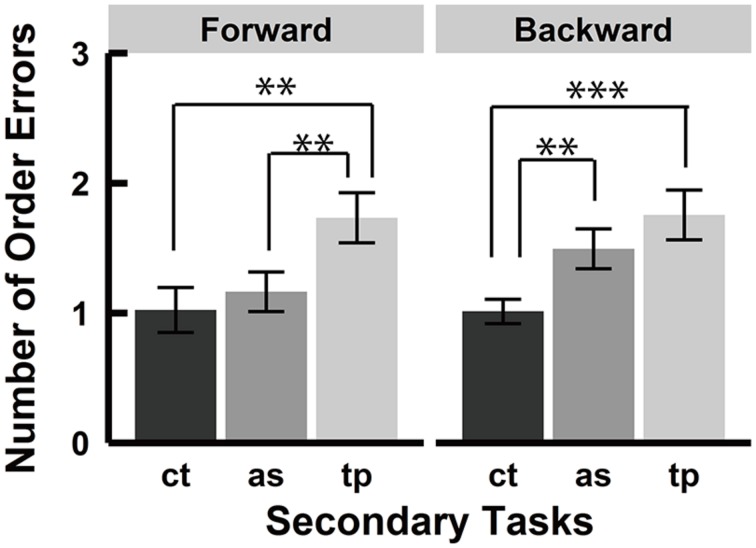
**Number of order errors.** Averages number of order errors in each secondary task. There was a significant main effect of the secondary task and significant differences between each secondary task. Although there was no significant interaction between the recall order and the secondary tasks, multiple comparisons showed that there were more order errors in the spatial tapping condition than the control condition in both the forward and backward conditions, while the serial articulatory suppression affected number of order errors in the backward condition only. (**p < 0.01, ***p < 0.001). ct, control condition; as, serial articulatory suppression condition; tp, spatial tapping condition.

**Figure 6** shows the average number of order errors in each condition. Analysis of the order errors revealed a significant main effect of secondary tasks [*F*(1.89, 33.96) = 30.84, *p* < 0.001, $\eta_{p}^{2}$ = 0.63] but no significant main effect of recall order [*F*(1, 18) = 0.77, *p* = 0.39, $\eta_{p}^{2}$ = 0.04] and no interaction between the recall order and the secondary tasks [*F*(1.96, 35.27) = 0.98, *p* = 0.38, $\eta_{p}^{2}$ = 0.05], which is inconsistent with experiment 1. Multiple comparisons for the secondary tasks’ effect showed that there were significant differences between the control and spatial tapping conditions (*p* < 0.001, *r* = 0.86), the serial articulatory suppression and spatial tapping conditions (*p* < 0.001, *r* = 0.76), and the control and serial articulatory suppression conditions (*p* < 0.01, *r* = 0.62). There was no significant interaction between the recall order and secondary task, which is inconsistent with the result of experiment 1. Although the interaction was not significant, we examined a simple main effect of the secondary task in the forward and backward condition separately. This analysis was performed to test whether serial articulatory suppression showed stronger interference with backward recall but not with forward recall, which was found in experiment 1. The analyses revealed that there were significant main effects of secondary tasks in the forward condition [*F*(1.96, 35.3) = 9.30, *p* < 0.001, $\eta_{p}^{2}$ = 0.34] and in the backward condition [*F*(1.94, 34.92) = 12.47, *p* < 0.001, $\eta_{p}^{2}$ = 0.41]. Multiple comparisons showed that there were significant differences between the control and spatial tapping conditions (*p* < 0.01, *r* = 0.67) and between the serial articulatory suppression and spatial tapping conditions (*p* < 0.01, *r* = 0.63), but no significant difference between the control and serial articulatory suppression conditions (*p* = 0.42, *r* = 0.19) in the forward condition. There were significant differences between the control and spatial tapping conditions (*p* < 0.001, *r* = 0.73) and between the control and serial articulatory suppression conditions (*p* < 0.01, *r* = 0.63), but no significant difference between the serial articulatory suppression and spatial tapping (*p* = 0.09, *r* = 0.39) in the backward condition. Therefore, serial articulatory suppression produced similar effects on order errors in both experiment 1 and 2.

## DISCUSSION

In experiment 2, we used a modified Corsi blocks task to prevent verbal strategies and investigated the effects of the serial articulatory suppression on forward and backward recall. Although the significant interaction with order error found in experiment 1 was not obtained, multiple comparisons analysis showed a consistent result, where the number of order errors was greater in the serial articulatory suppression condition than in the control condition in the backward condition, while no such difference was found in the forward recall condition. It should be noted that performance of the secondary tasks did not differ between the forward and backward conditions. These results indicate that spatial and sequential interference were equivalent in the forward and backward conditions.

### CORRECT TRIALS

Both spatial tapping and serial articulatory suppression decreased the number of correct trials in both the forward and backward conditions. This result is inconsistent with that from experiment 1, where only spatial tapping decreased the number of correct trials. The inconsistency of the results might be due to a difference in the task difficulty, where a random layout of block positions increased the overall task difficulty, however, the number of correct trials appeared equivalent in the two experiments (*M* = 4.17, SD = 1.74, in the control condition at sequence length 7 in experiment 1, and *M* = 4.32, SD = 1.44, in the control condition in experiment 2). Another possible explanation why the serial articulatory suppression decreased the number of correct trials was that both forward and backward recall may have required stronger sequential representation when the layout of block positions was varied across trials. In other words, a fixed layout of the block positions might help bind order representation to mask the effect of the serial articulatory suppression on the number of correct recalls in experiment 1. This theory requires further investigation.

### POSITION ERROR AND ORDER ERROR

Positions error was not affected by the secondary tasks, which disagrees with the findings in experiment 1. We discuss possible reasons for the discrepancy below.

Regarding order error, both spatial tapping and serial articulatory suppression increased the number of the errors in the forward and backward conditions. This result is inconsistent with that in experiment 1, where the serial articulatory suppression selectively increased order errors in backward recall. As described above, the main effect of the secondary tasks on recall order might have resulted from the varied layout of the block positions, which might have demanded more sequential processing compared to a fixed layout. Although an interaction between the factors of recall order and secondary task was insignificant, we tested the hypothesis retrieved from the result of experiment 1: greater dependency on sequential processing in the backward condition. For this purpose, we separately analyzed order errors in the forward and backward conditions and found that the number of order errors was greater in the serial articulatory suppression condition than in the control condition in the backward condition, while such a difference was not observed in the forward recall. This pattern is consistent across the two experiments, although a disappearance of the interaction between the factors of recall order and secondary tasks must be noted.

## GENERAL DISCUSSION

The present study aimed to specify cognitive processes that dissociated forward and backward recall in the Corsi blocks task. Focusing attention on spatial and sequential processing, we investigated which process plays more critical roles in forward and backward recall. Two experiments showed that spatial tapping, which disturbs spatial processing, affected position error in forward and backward recall equally. This result indicates that forward and backward recall depend on spatial processing to a similar extent. On the other hand, serial articulatory suppression, which is thought to disrupt order coding, increased the number of order errors in backward recall. This result supports the hypothesis that sequential processing is the critical factor that dissociates forward and backward recall in the Corsi blocks task. In other words, stronger order coding may be required to rearrange the sequence of spatial positions of blocks in the reverse order.

Measuring the cognitive ability of visuospatial learning disabled children, Mammarella and Cornoldi (2005) showed that those children had difficulty in performing backward recall in the Corsi blocks task but performed normally in backward recall of the digit span task. This result led the authors to conclude that those children’s executive function was preserved. The result also can imply, however, that backward recall in the Corsi blocks task requires less executive function and more spatial processing. Vandierendonck et al. (2004) found that disruption of spatial processing by spatial tapping affected both forward and backward recall similarly, suggesting that the two recalls similarly recruit spatial processing. Consistent with that finding, the present study showed equivalent effects of spatial tapping on forward and backward recall. In addition, we analyzed position errors, which are more sensitive to interference from spatial processing. These errors were counted when participants selected blocks that had not been highlighted in the encoding. In experiment 1, position errors were similarly increased by spatial tapping in both the forward and backward conditions. This result further supports the idea that forward and backward recalls depend on spatial processing to a similar extent. In experiment 2, however, spatial tapping did not increase position errors, although the spatial positions of blocks were varied across trials. This result is opposite our expectation, which was that a varied layout of placeholders would increase spatial errors and spatial tapping would have accentuated this effect. One possible account of the null result was discriminability of the block positions. Because we placed blocks spaced by a certain distance in order to avoid overlapping, the procedure made it easier for participants to discriminate the positions. In fact, the number of spatial errors was slightly smaller in experiment 2 than in experiment 1.

Sequential processing is another cognitive process critically involved in the Corsi blocks task. Some studies have proposed a modality-independent system for order coding (e.g., Jones et al., 1995; Depoorter and Vandierendonck, 2009). Devoting a special attention to the system, we measured the number of order errors under concurrent interference tasks. Especially, we were interested in serial articulatory suppression, which was shown to impair order coding in both verbal and visuospatial materials. We found that order errors in the backward condition were greater in the serial articulatory suppression condition than in the control condition, but no difference in the forward condition in experiment 1. However, we cannot exclude the possibility that participants might have employed verbal coding of the sequential information by naming each block (“top right,” “left bottom,” and “so on”), as block positions were fixed across trials. Varying the positions of blocks across trials, we examined the effects of secondary tasks in experiment 2. If participants had employed a verbal coding strategy, the serial articulatory suppression would have no longer affected task performance. In contrast to experiment 1, where an interaction between the recall order and the secondary tasks was obtained, a significant main effect of the secondary task was observed in experiment 2 and following multiple comparisons yielded a greater number of order errors in the serial articulatory suppression condition than in the control condition. Therefore, the result indicates that the serial articulatory suppression affected both forward and backward recall rather than losing its selective effect on backward recall. One possible explanation for this result was that varying the block positions demanded stronger order representation not only in backward recall but also forward recall. However, when performing multiple comparisons over the non-significant interaction data, order error in the backward condition was greater in the serial articulatory suppression condition than in the control condition, while the number of the errors did not differ in the forward condition, which is consistent with the results in experiment 1. These results support the hypothesis that sequential processing differentiates forward recall from backward recall in the Corsi blocks task. Specifically, backward recall would strongly depend on a modality-independent order coding system in order to make stronger order representation, which would be required to rearrange a sequence of spatial positions of blocks in the reverse order.

It should be noted that spatial tapping increased the number of order errors in both the forward and backward conditions across the two experiments. Our interpretation of this observation is that the spatial tapping interfered with the integration of visuospatial and order information, but less so the order coding itself. In fact, the matrix tapping employed in the present study (tapping four corners of a square) is thought to be less effective to order coding (Depoorter and Vandierendonck, 2009).

It should be noted that time differences between the forward and backward conditions may have contributed to the different error rates. For example, in the forward condition, the time lag between the first item being encoded and retrieved was six items, whereas in the backward condition it was twelve items. Furthermore, despite having nine blocks on the screen, we only used seven blocks in most of our experiments. Therefore, position error could not be well evaluated compared with order error because in a sequence length of seven items, the number of position errors is at most two. Using more blocks as distractions may increase the position error. However, it should be noted that the standard Corsi blocks task uses nine blocks.

There is an opinion that using the backward Corsi blocks task and similar spatial span task for clinical purposes or the assessment of working memory is insignificant, because many studies did not find behavioral differences between forward and backward recall (Kessels et al., 2008). However, our research has shown that there is a difference between forward and backward recall in the Corsi blocks task for serial order memory. Thus, other behavioral indices in the task (i.e., order errors) can be used to assess individual sequential spatial function.

## CONCLUSION

The present study provided evidence that sequential processing is the critical cognitive factor that dissociates forward recall from backward recall in the Corsi blocks task. This conclusion was drawn by the finding that serial articulatory suppression, which is thought to interfere with the modality-independent order coding system, selectively impaired backward recall. The present finding may need to be considered when using the Corsi blocks task to measure individual visuospatial ability as part of an intellectual test.

## Conflict of Interest Statement

The authors declare that the research was conducted in the absence of any commercial or financial relationships that could be construed as a potential conflict of interest.
